# Radiology domain in the diagnosis of IgG4-RD according to the 2019 American College of Rheumatology and European League Against Rheumatism classification

**DOI:** 10.1186/s13244-024-01638-3

**Published:** 2024-03-26

**Authors:** Khaled Y. Elbanna, Jie-Ying Kowa, Nikhil Mirajkar, Korosh Khalili, Tae Kyoung Kim

**Affiliations:** University Medical Imaging Toronto, University Health Network, Toronto, ON Canada

**Keywords:** Immunoglobulin G4-related disease, Classification, Radiology

## Abstract

**Objectives:**

To evaluate the performance of radiology-related inclusion criteria of the 2019 ACR-EULAR classification system in the diagnosis of IgG4-related disease (IgG4-RD).

**Methods:**

This retrospective single-institution study included patients who received a diagnosis of IgG4-RD between January 2010 and December 2020. Two abdominal radiologists independently reviewed baseline imaging studies and scored radiology findings according to the 2019 ACR-EULAR classification criteria. Additional scores were assigned based on serological, histopathological, and immunostaining features.

**Results:**

Seventy-four patients (58 males and 16 females) with a mean age of 59.3 ± 13.9 years diagnosed with IgG4-RD were included. 51/74 (68.9%) were classified as having IgG4-RD according to the 2019 ACR-EULAR classification criteria. To reach a score ≥ 20 in these 51 patients, the radiology domain was sufficient in 20/51 (39.2%) and adding the serology domain was required for another 20/51 (39.2%). The remaining 11/51 patients (21.6%) required the histopathology and immunostaining domains. Radiological involvement of two or more organs at presentation was significantly associated with a score of ≥ 20 and seen in 43/51 (84.3%) compared to 5/23 (21.7%) of the non-classified group (*p* < 0.001). The group classified as having IgG4-RD showed a significantly higher proportion of elevated IgG4 levels (39/51, 76.5%) than the non-classified group (8/23, 34.8%) (< 0.001).

**Conclusion:**

The study findings support the effectiveness of the radiology-related inclusion criteria of the 2019 ACR-EULAR classification system in diagnosing IgG4-RD. Combining radiology and serology domains achieved the cut-off in 80% of IgG-RD patients, enabling non-invasive diagnosis. The classification of IgG4-RD was significantly associated with multi-organ involvement, particularly affecting the pancreas and biliary system.

**Critical relevance statement:**

This study is the first to evaluate the diagnostic performance of the radiology domain in the 2019 ACR-EULAR classification criteria. The study results confirm its utility and potential to enable non-invasive diagnosis when combined with serological testing in a significant proportion of patients.

**Key points:**

• A significant proportion of patients can be diagnosed with IgG4-RD using the radiology and serology domains exclusively.

• Multi-organ involvement is significantly associated with classifying patients as IgG4-RD, with the pancreas and biliary system most frequently affected.

• A high level of inter-reader agreement in the scoring of the radiology domain supports its reliability.

**Graphical Abstract:**

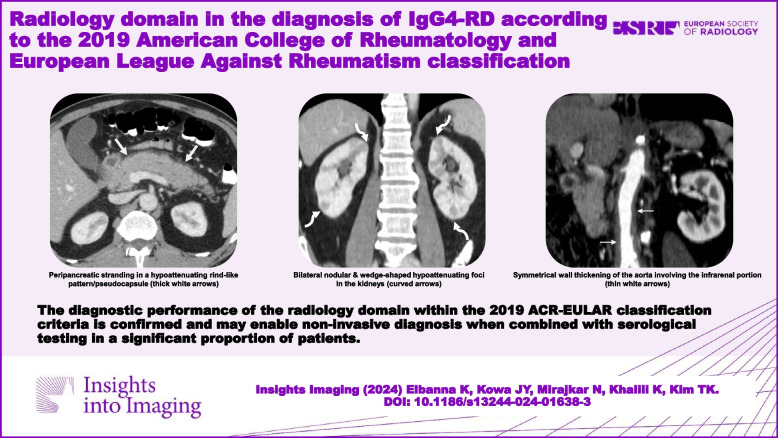

## Introduction

In clinical practice, diagnosing Immunoglobulin G4-related disease necessitates a comprehensive approach that integrates clinical, serological, radiological, and histopathological findings. The distinctive histopathologic features, including lymphoplasmacytic infiltrates, fibrosis, and obliterative phlebitis, play a pivotal role in the diagnosis of IgG4-RD [1]. However, obtaining tissue biopsies from specific organs can be challenging. Moreover, the variation in serum IgG4 levels and the presence of nonspecific radiological findings add to the complexities in diagnosing IgG4-RD [2]. Rigorous clinical-pathological correlation is imperative due to the  the disease's diverse clinical manifestations, which can resemble both malignant and inflammatory conditions. Multiple diagnostic criteria have been formulated by various research groups to facilitate a precise diagnosis of IgG4-RD, incorporating imaging, histologic and serologic findings, extrapancreatic involvement, and response to corticosteroid therapy [3–8].

In 2019, the American College of Rheumatology and European League Against Rheumatism (ACR-EULAR) released classification criteria to improve the recognition of IgG4-RD and to distinguish this uncommon condition from disease mimickers. The ACR-EULAR investigators reported excellent specificity of 97.8% and moderately high sensitivity of 82.0% of  the classification algorithm when applied to an international validation cohort [9, 10]. Other groups have evaluated the criteria in real-world settings and confirmed its high specificity [11–15].

ACR/EULAR has proposed a three-step classification process comprising: (a) clinical, radiological or pathological criteria required to enter the classification algorithm; (b) a set of exclusion criteria eliminating the patient from further IgG4-RD classification; and (c) a set of numerically weighted inclusion criteria that include clinical, serological, radiological, and pathological findings. The patient is classified as IgG4-RD if the cumulative score of these inclusion criteria equals 20 points or more [9, 10].

The 2019 ACR-EULAR classification criteria allow patients to be classified accurately even without biopsy or elevated serum IgG4 level [9, 10], potentially overcoming some of the diagnostic challenges encountered in routine clinical practice. As there is no single defining feature of IgG4-RD, accurate classification integrates evidence across clinical, serological, histopathological, and radiology domains. Of note, radiology findings account for 5 out of 8 scoring domains in the 2019 ACR-EULAR classification criteria. Ren et al. have provided a review on imaging interpretation per classification criteria in clinical practice [16] but, to our knowledge, there has yet to be published studies evaluating the diagnostic performance of the radiology domain.

We sought to investigate the diagnostic performance of radiology inclusion criteria proposed by ACR-EULAR in a real-world cohort of patients referred to our tertiary care centre with a suspected diagnosis of IgG4-RD.

## Methods

### Patient selection

This HIPAA-compliant retrospective study was REB-approved, and informed consent was waived.

To retrieve patients referred to our radiology department with IgG4-RD, our Radiology Information System (RIS) was queried over a 10-year period from January 2010 to December 2020 for the following keywords: “IgG4-RD”, “IgG4”, “autoimmune pancreatitis”, and “autoimmune sclerosing cholangitis”.

The medical records were initially reviewed to exclude patients per exclusion criteria proposed by the ACR-EULAR [9, 10] and those with only elevated serum IgG4 levels but without clinical or radiological evidence of the disease. We enrolled patients with a confirmed diagnosis of IgG4-RD according to the 2020 Revised Comprehensive Diagnostic criteria [8] and continued to be managed as such without an alternative diagnosis. The medical records of the enrolled patients were reviewed, and demographic information (sex, age at diagnosis) and serological and histopathology results were recorded. Table 1 summarises the ACR-EULAR entry and exclusion criteria [9, 10].

**Table 1:** Entry and exclusion items of the 2019 ACR-EULAR classification criteria [1, 2]

**Entry criteria**	
*Clinical or radiological*	Characteristic finding of enlargement/tumour-like mass in a typical organ (e.g. pancreas, salivary glands, orbits, kidney, retroperitoneum, pachymeninges or thyroid gland) *Exceptions:* (i) Bile ducts—narrowing (ii) Aorta—wall thickening or aneurysmal dilatation (iii) Lungs—thickening of the bronchovascular bundles
*Pathological*	An inflammatory process accompanied by a lymphoplasmacytic infiltrate in one of the organs above
**Exclusion criteria**	
*Clinical*	Fever Lack of response to glucocorticoids
*Serological*	Unexplained leukopenia and thrombocytopenia Peripheral eosinophilia Positive antineutrophil cytoplasmic antibody (against proteinase 3 or myeloperoxidase) Positive SSA/Ro or SSB/La antibody Positive double-stranded DNA, ribonucleoprotein or Smith antibody Other disease-specific autoantibodies Cryoglobulinemia
*Radiological*	Findings suggesting malignancy or infection Rapid progression Long bone abnormalities consistent with Erdheim-Chester disease Splenomegaly
*Pathological*	Cellular infiltrates suggesting malignancy Markers of inflammatory myofibroblastic tumour Prominent neutrophilic infiltration Necrotising vasculitis Prominent necrosis Primarily granulomatous inflammation Features of macrophage or histiocytic disorder
*Existing diagnoses*	Multicentric Castleman’s disease Crohn’s disease or ulcerative colitis (if isolated pancreaticobiliary disease) Hashimoto thyroiditis (if isolated thyroid involvement)

### Imaging review and radiology domain score

In this study, two radiologists, one experienced radiologist with 8 years of experience in abdominal imaging and an abdominal imaging fellow, were tasked with independently and retrospectively reviewing baseline imaging studies including CT, MRI, and US to generate scores for the radiology domain. While both readers were aware of the IgG4-RD diagnosis in the cohort, they were blinded to imaging findings in existing radiology reports. Before scoring the imaging findings, the radiologists convened to review the ACR-EULAR inclusion criteria definitions, and established supplementary guidelines for imaging interpretation to improve consistency in scoring (Table 2). Each radiologist independently recorded the numerical value of the imaging findings for each anatomical region/organ. A consensus was then obtained between the two radiologists for discrepant reads.

**Table 2:** Supplementary guidelines for image interpretation [3, 11, 15, 16]

**Organs**	**Typical imaging findings**
*Lacrimal, parotid, sublingual and submandibular glands*	US: heterogeneous, hypoechoic enlargement CT: gland enlargement on contrast-enhanced CT MRI: gland enlargement with low T1/low T2 signal intensity
*Chest*	CT: peribronchovascular and septal thickening CT: paravertebral band-like soft tissue which is asymmetrical, does not encase the aorta, and is usually right-sided (T8-T11 level)
*Pancreaticobiliary*	Diffuse pancreatic enlargement (involves > 2/3 of the pancreas) and irregular narrowing of the main pancreatic duct • CT: hypoattenuating on pancreatic phase with delayed enhancement • MRI: low T1/high T2 signal intensity, restricted diffusion on diffusion wighted imaging, and delayed enhancement Capsule-like rim: a low-attenuation rim on contrast enhanced CT or a low T1/T2 signal intensity rim on MRI surrounding all or part of the pancreas Typical biliary involvement: proximal bile ducts show smooth wall thickening and can be identified on US, CT, or MR
*Kidney*	Uroepithelial thickening or soft tissue involving the renal pelvis identified on CT or MRI Round or wedge-shaped renal cortical lesions • CT: hypoattenuating on corticomedullary phase, progressive enhancement on nephrographic phase • MRI: low T1/variable T2 signal intensity, low intensity on corticomedullary phase and progressive enhancement on nephrographic phase
*Retroperitoneum*	Aortic wall thickening or periaortic soft tissue • CT: isoattenuating to muscle • MR: low to isointense T1/T2 signal intensity with homogeneous delayed enhancement

The points scored for the radiology domain were added to those scored by other weighted inclusion criteria, collected from the medical records, including histopathology, immunostaining and serum IgG4, as shown in Table 3. Patients with a total score of ≥ 20 points were classified as IgG4-RD according to the 2019 ACR-EULAR classification criteria.

**Table 3:** A scoring system of the 2019 ACR-EULAR classification criteria [1, 2]

**Domains**	**Weighted items**	**Score**
*Histopathology*	(i) Uninformative biopsy	+ 0
	(ii) Dense lymphocytic infiltration	+ 4
	(iii) Dense lymphocytic infiltration and obliterative phlebitis	+ 6
	(iv) Dense lymphocytic infiltration and storiform fibrosis	+ 13
*Immunostaining*	(i) IgG4 + :IgG + ratio 0–40% (or indeterminate) & number of IgG4 + cells/HPF 0–9	+ 0
	(ii) IgG4 + :IgG + ratio ≥ 41% & number of IgG4 + cells/HPF 0–9 (or indeterminate), OR IgG4 + :IgG + ratio 0–40% (or indeterminate) & number of IgG4 + cells/HPF ≥ 10 (or indeterminate)	+ 7
	(iii) IgG4 + :IgG + ratio 41–70% & number of IgG4 + cells/HPF ≥ 10, OR IgG4 + :IgG + ratio ≥ 71% & number of IgG4 + cells/HPF 10–50	+ 14
	(iv) IgG4 + :IgG + ratio ≥ 71% & number of IgG4 + cells/HPF ≥ 51	+ 16
*Serum IgG4 concentration*	(i) Normal/not checked	+ 0
	(ii) > Normal but < 2 × ULN	+ 4
	(iii) 2–5 × ULN	+ 6
	(iv) ≥ 5 × ULN	+ 11
*Lacrimal, parotid, sublingual and submandibular glands*	(i) No pair of glands involved	+ 0
	(ii) One pair involved	+ 6
	(iii) Two or more pairs involved	+ 14
*Chest*	(i) No evidence of typical involvement	+ 0
	(ii) Peribronchovascular and septal thickening	+ 4
	(iii) Paravertebral band-like soft tissue	+ 10
*Pancreatobiliary*	(i) No evidence of typical involvement	+ 0
	(ii) Diffuse pancreatic enlargement	+ 8
	(iii) Diffuse pancreatic enlargement & capsule-like rim	+ 11
	(iv) Pancreatic involvement (either ii or iii) & biliary involvement	+ 19
*Kidney*	(i) No evidence of typical involvement	+ 0
	(ii) Hypocomplementemia	+ 6
	(iii) Renal pelvis thickening/soft tissue	+ 8
	(iv) Bilateral patchy/round cortical low-density areas	+ 10
*Retroperitoneum*	(i) No evidence of typical involvement	+ 0
	(ii) Diffuse thickening of the abdominal aortic wall	+ 4
	(iii) Circumferential/anterolateral soft tissue around infrarenal aorta or iliac arteries	+ 8

Only the highest-weighted item in each subdomain is scored

A case meets the 2019 ACR-EULAR classification criteria with a score of 20 points or more

### Statistical analysis

Descriptive statistics were used for patient demographics and performance of the 2019 ACR-EULAR classification criteria. Categorical variables are presented as numbers and percentages. Continuous variables are presented as the mean (± standard deviation, SD), median, and interquartile range (IQR). Differences were evaluated using Fisher’s exact test for categorical variables and the Mann-Whitney *U* test for continuous variables. A *p*-value less than 0.05 was considered statistically significant. Agreement between readers was quantified with either Cohen’s unweighted or Fleiss’ weighted kappa statistics with a 95% confidence interval (> 0.80 was considered almost perfect, 0.61–0.80 substantial, 0.41–0.60 moderate and < 0.40 poor reliability).

## Results

### Patient characteristics

Initially, a search of our RIS database revealed 276 patients. After applying exclusion criteria, 74 patients (58 males and 16 females) with a mean age of 59.3 ± 13.9 years who had been diagnosed with IgG4-RD, were included in the study. We excluded 109 patients who had an alternative final diagnosis, including primary sclerosing cholangitis (*n* = 35), pancreatic ductal adenocarcinoma (*n* = 18), lymphoma (*n* = 12), cholangiocarcinoma (*n* = 10), pancreatitis (*n* = 8), retroperitoneal fibrosis (*n* = 5), vasculitis (*n* = 4), Erdheim-Chester disease (*n* = 3), and others (*n* = 14). Also, per the 2019 ACR-EULAR exclusion criteria, three patients were excluded due to a coexisting diagnosis of inflammatory bowel disease. Additionally, 90 patients were excluded, as they were referred to imaging due to elevated serum IgG4 levels, but no clinical or radiological evidence of the disease was found.

### Review and scoring of radiology, serology and pathology domains

The radiologists reviewed a total of 112 baseline imaging studies, including CT Abdomen (*n* = 55), MRI Abdomen (*n* = 19), CT Chest (*n* = 14), US Head and Neck (*n* = 11), CT Head and Neck (*n* = 7), MRI Head and Neck (*n* = 5), and US Abdomen (*n* = 1). Table 4 displays readers’ scores for each anatomical region and highlights the inter-reader agreement of radiological domain scoring across different anatomical regions. The interobserver agreement was almost perfect in scoring radiology domains of the abdominal organs and salivary and lacrimal glands and substantial in the assessment of the chest findings. The overall radiology domain score per patient was evaluated, with a score of ≥ 20 achieved in 20/74 (27.0%) and 18/74 (24.3%) of patients per reader 1 and reader 2, respectively. The inter-reader agreement was 0.863 (95% CI: 0.733 to 0.993).

**Table 4:** Inter-reader agreement of radiology domain scoring across different anatomical regions/organs

**Score categories**	**Reader 1**	**Reader 2**	**Inter-reader agreement (95% CI)**
**Lacrimal and salivary glands**			
+ *0 points*	59/74 (79.7%)	61/74 (82.4%)	0.917 (0.803, 1)
+ *6 points*	9/74 (12.2%)	8/74 (10.8%)	
+ *14 points*	6/74 (8.1%)	5/74 (6.8%)	
**Chest**			
+ *0 points*	68/74 (91.8%)	71/74 (95.9%)	0.653 (0.290, 1)
+ *4 points*	3/74 (4.1%)	2/74 (2.7%)	
+ *10 points*	3/74 (4.1%)	1/74 (1.4%)	
**Pancreaticobiliary**			
+ *0 points*	26/74 (35.1%)	26/74 (35.1%)	0.956 (0.897, 1)
+ *8 points*	1/74 (1.4%)	1/74 (1.4%)	
+ *11 points*	10/74 (13.5%)	12/74 (16.2%)	
+ *19 points*	37/74 (50%)	35/74 (47.3%)	
**Kidney**			
+ *0 points*	55/74 (74.3%)	55/74 (74.3%)	0.967 (0.903, 1)
+ *8 points*	4/74 (5.4%)	5/74 (6.8%)	
+ *10 points*	15/74 (20.3%)	14/74 (18.9%)	
**Retroperitoneum**			
+ 0 points	59/74 (79.7%)	60/74 (81.0%)	0.960 (0.881, 1)
+ 4 points	8/74 (10.8%)	7/74 (9.5%)	
+ 8 points	7/74 (9.5%)	7/74 (9.5%)	

Percentages of the total number are expressed in brackets for each variable. The unweighted Cohen’s kappa statistic is used to express agreement

**Fig. 1:**
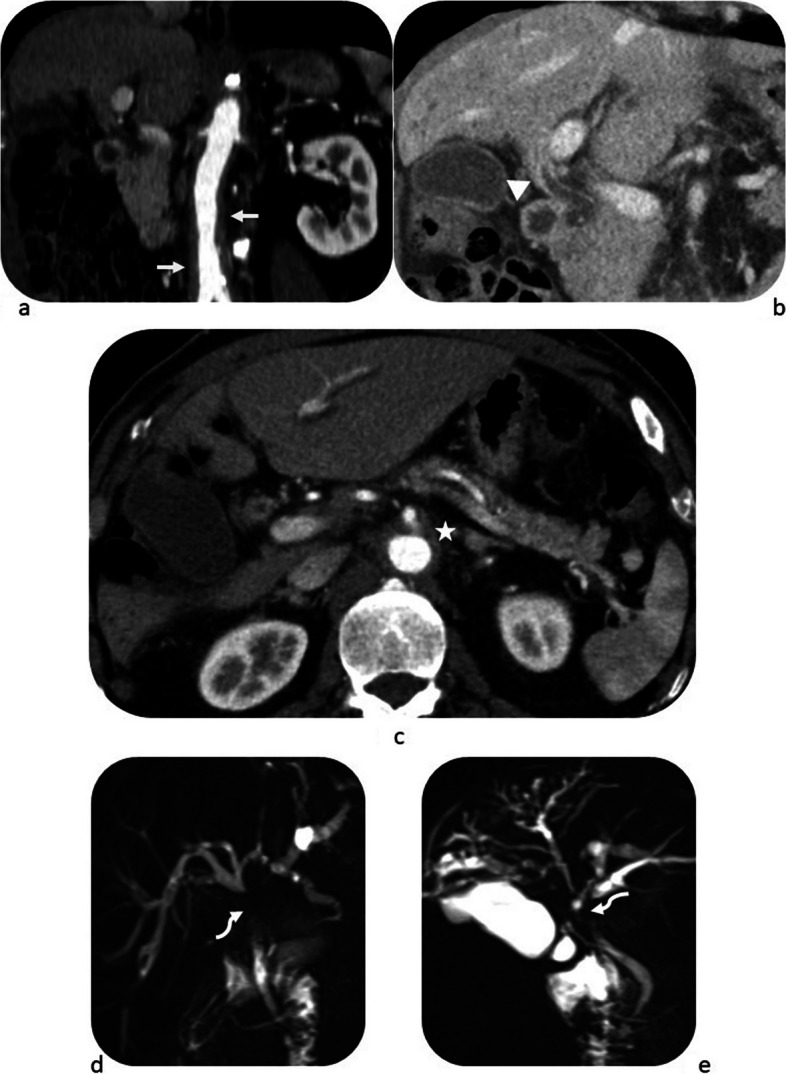
Case of pancreaticobiliary involvement scoring 19, periaortitis scoring 4 points and elevated serum IgG_4_ scoring 4 points. Overall score = 27 points. **a** Coronal reformat arterial phase CT image shows symmetrical wall thickening of the aorta involving the infrarenal aorta (white arrows). **b** Coronal reformat portal venous CT image shows mural thickening and hyperenhancement of the CBD wall (white arrowhead). **c** Axial arterial phase CT image shows concentric wall thickening of the aorta in keeping with periaortitis (white star). **d**, **e** Two views of coronal MRCP show a stricture involving the CBD over a 3-cm segment (curved arrows), with upstream intrahepatic biliary duct dilatation

**Fig. 2:**
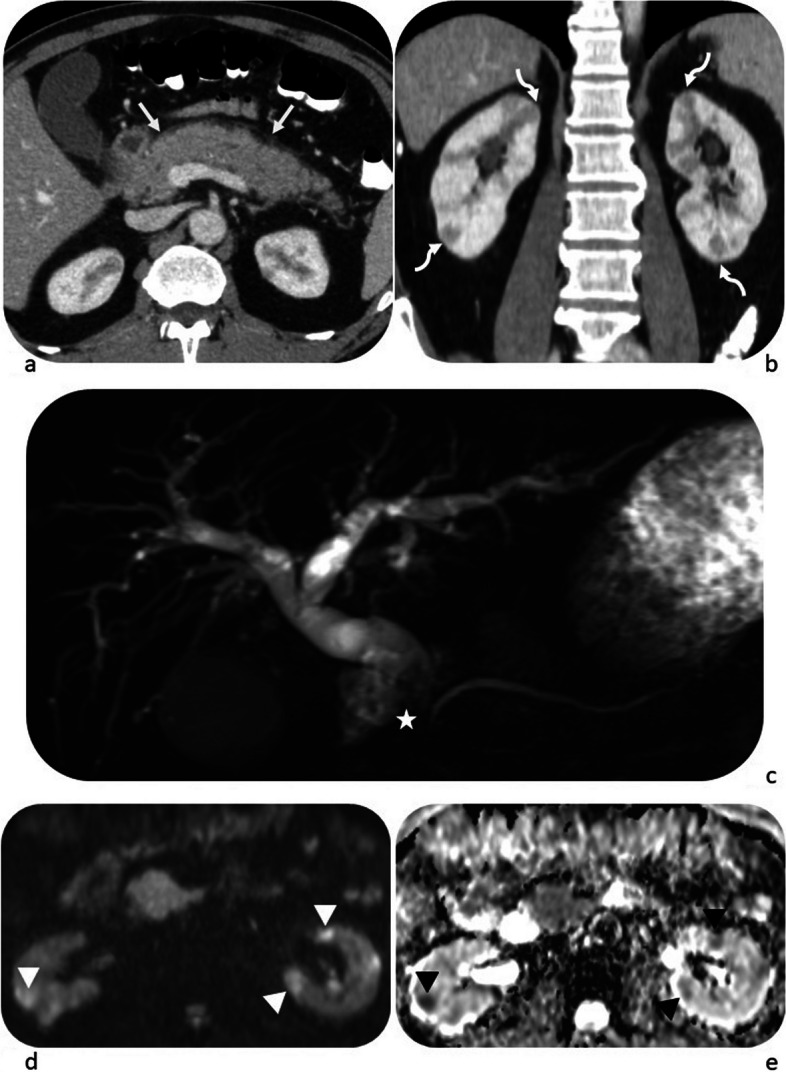
Case of pancreaticobiliary involvement scoring 19 points, bilateral renal involvement scoring 10 points and a 5-fold increase above the normal limit for serum IgG_4_ scoring 11 points. Overall score = 40 points. **a** Axial portal venous phase CT image shows diffuse pancreatic enlargement, with peripancreatic stranding in a hypoattenuating rind-like pattern/pseudocapsule (white arrows). **b** Coronal reformat portal venous CT image shows multiple bilateral nodular and wedge-shaped hypoattenuating lesions (curved arrows). **c** Coronal MRCP image shows dilation of the intra- and extra-hepatic bile ducts down to a smooth narrowing of the distal CBD over 2-cm segment (white star). The pancreatic duct is smooth and of a normal calibre. **d**, **e** Axial DWI & ADC map images show multifocal areas of restricted diffusion in both kidneys (arrowheads)

**Fig. 3:**
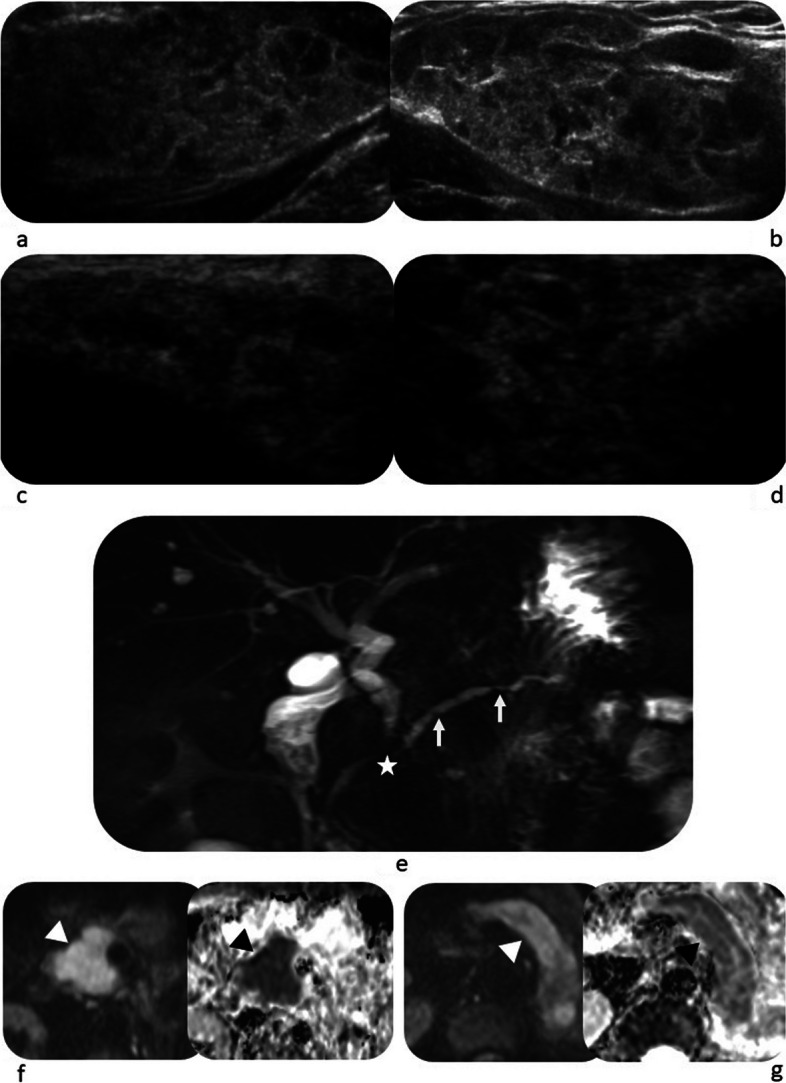
Case of bilateral submandibular and lacrimal gland involvement scoring 14 points, pancreaticobiliary involvement scoring 19 points, biopsy of submandibular glands revealed dense lymphocytic infiltration scoring 4 points with positivity on immunostaining scoring 7 points and elevated serum IgG_4_ scoring 11 points. Overall score = 55 points. **a** and **b** Ultrasound images of the left and right submandibular glands, respectively, show bilateral enlargement of the submandibular glands, with heterogenous echotexture and multiple hypoechoic nodules. **c** and **d** Ultrasound images of the left and right lacrimal glands, respectively, show multiple hypoechoic nodules in both lacrimal glands. **e** Coronal MRCP image shows mildly dilated main pancreatic duct with multifocal narrowing and irregularity (white arrows), and segmental narrowing of the intrapancreatic CBD (white star) causing mild upstream dilatation. **f**, **g** Axial DWI & ADC map images show restricted diffusion involving the pancreatic head and body (arrowheads) with relative sparing of the tail

After the consensus review by two radiologists and collective evaluation of all domains (radiology, serology, histopathology and immunostaining), 51/74 (68.9%) of patients were classified as having IgG4-RD (Table 5). In these 51 patients, the radiology domain alone was sufficient to achieve a score of 20 or more in 20/51 patients (39.2%). However, in another 20/51 (39.2%) patients, the serology domain had to be added to achieve the cut-off score. The remaining 11/51 (21.6%) patients required the histopathology and immunostaining domains to be classified as IgG4-RD. It was observed that patients who presented with radiological involvement of two or more organs were significantly more likely to have a score of 20 or higher. This was seen in 43/51 (84.3%) patients who were classified as having IgG4-RD, as compared to 5/23 (21.7%) patients in the non-classified group (*p* < 0.001). The most commonly affected organs were the pancreas and biliary system, observed in 40 and 33 patients, respectively (Table 5). Figures 1, 2 and 3 provide examples for radiology domain scoring.

**Table 5:** Scoring of radiology, serology, histopathology and immunostaining domains in all 74 patients

Radiology domain score							Other domain score			All domain score	Meeting criteria
Pancreas	Biliary	Kidney	Lacrimal/salivary	Retroperitoneal	Chest	Score	Serology	Histopathology	Immunostaining		
+	+	+	+	−	+	47	0	0	0	47	+
+	+	+	+	−	−	35	11	0	0	46	+
+	+	+	−	+	+	41	0	0	0	41	+
+	+	+	−	−	+	39	11	4	0	54	+
+	+	+	−	+	−	33	0	0	0	33	+
+	+	+	−	−	−	29	11	13	14	67	+
+	+	+	−	−	−	29	11	0	0	40	+
+	+	+	−	−	−	29	6	0	0	35	+
+	+	+	−	−	−	29	11	0	0	40	+
+	+	+	−	−	−	29	0	0	0	29	+
+	+	+	−	−	−	29	6	0	14	49	+
+	+	−	+	−	−	33	11	4	7	55	+
+	+	−	−	−	+	23	0	0	7	30	+
+	+	−	−	+	−	23	11	0	0	34	+
+	+	−	−	+	−	23	11	0	0	34	+
+	+	−	−	+	−	23	4	0	0	27	+
+	+	−	−	−	−	19	0	13	7	39	+
+	+	−	−	−	−	19	4	0	0	23	+
+	+	−	−	−	−	19	6	0	14	39	+
+	+	−	−	−	−	19	11	0	0	30	+
+	+	−	−	−	−	19	0	4	7	30	+
+	+	−	−	−	−	19	6	0	0	25	+
+	+	−	−	−	−	19	4	0	0	23	+
+	+	−	−	−	−	19	6	0	0	25	+
+	+	−	−	−	−	19	11	0	0	30	+
+	+	−	−	−	−	19	11	4	7	41	+
+	+	−	−	−	−	19	6	0	0	25	+
+	+	−	−	−	−	19	6	0	0	25	+
+	+	−	−	−	−	19	6	0	0	25	+
+	+	−	−	−	−	19	6	0	0	25	+
+	+	−	−	−	−	19	6	0	0	25	+
+	+	−	−	−	−	19	6	0	0	25	+
+	+	−	−	−	−	19	0	13	7	39	+
+	−	+	+	−	−	32	11	4	14	61	+
+	−	+	−	+	−	27	0	0	7	34	+
+	−	+	−	+	−	25	11	13	14	63	+
+	−	−	+	−	−	17	0	0	7	24	+
+	−	−	+	−	−	17	6	4	0	27	+
+	−	−	−	+	−	19	6	0	0	25	+
+	−	−	−	−	−	11	11	0	0	22	+
**−**	+	+	+	+	−	28	11	0	0	39	+
**−**	+	+	−	−	−	8	6	0	14	28	+
**−**	−	+	+	−	−	16	0	4	14	34	+
**−**	−	+	−	+	−	18	11	0	0	29	+
**−**	−	+	−	−	−	8	11	6	0	25	+
**−**	−	−	+	−	−	14	0	0	7	21	+
**−**	−	−	+	−	−	6	11	6	14	37	+
**−**	−	−	+	−	−	14	11	0	0	25	+
**−**	−	−	+	−	−	6	6	4	7	23	+
**−**	−	−	−	+	+	18	6	13	7	44	+
**−**	−	−	−	+	−	8	4	4	7	23	+
+	+	−	−	−	−	19	0	0	0	19	−
+	+	−	−	−	−	19	0	0	0	19	−
+	+	−	−	−	−	19	0	0	0	19	−
+	+	−	−	−	−	19	0	0	0	19	−
+	−	−	−	−	−	11	6	0	0	17	−
+	−	−	−	−	−	11	0	0	0	11	−
+	−	−	−	−	−	11	6	0	0	17	−
+	−	−	−	−	−	11	6	0	0	17	−
**−**	+	−	−	+	−	8	6	0	0	14	−
**−**	+	−	−	−	−	0	0	0	0	0	−
**−**	+	−	−	−	−	0	0	0	14	14	−
**−**	−	−	−	−	−	0	0	4	7	11	−
**−**	−	−	+	−	−	6	0	4	7	17	−
**−**	−	−	+	−	−	6	0	4	0	10	−
**−**	−	−	+	−	−	6	11	0	0	17	−
**−**	−	−	−	−	+	4	0	0	7	11	−
**−**	−	−	−	+	−	4	6	0	0	10	−
**−**	−	−	−	+	−	8	0	4	7	19	−
**−**	−	−	−	−	−	0	11	0	0	11	−
**−**	−	−	−	−	−	0	6	4	7	17	−
**−**	−	−	−	−	−	0	0	4	7	11	−
**−**	−	−	−	−	−	0	0	0	7	7	−
**−**	−	−	−	−	−	0	0	0	0	0	−

The group classified as having IgG4-RD showed a significantly higher proportion of elevated serum IgG4 levels (39/51, 76.5%, median: 356, IQR: 204–1580, range: 150–2330) than the non-classified group (8/23, 34.8%, median 291.5, IQR 276.5–496, range 263–609) (*p* < 0.001).

Biopsy results were available for 56.7% (42/74) of the patients; pancreas (*n* = 8), gallbladder (*n* = 1), kidney (*n* = 7), lymph nodes (*n* = 4), retroperitoneum (*n* = 4), lacrimal/orbital (*n* = 6), submandibular salivary glands (*n* = 6), leptomeninges (*n* = 1), nasopharynx (*n* = 1), liver (*n* = 3) and lung (*n* = 1). Among these 42 patients, 22 (52.3%) met the characteristic histopathological findings including 15 with a score of 4, two with a score of 6 and five with a score of 13 points. Immunostaining was positive in 28/42 (66.7%) patients including 19 with a score of 7 points and 9 with a score of 14 points. No significant difference was identified between sex or age and the classification outcome (Table 5).

## Discussion

This retrospective study aimed to assess the radiology domain performance in the 2019 ACR-EULAR classification criteria for IgG4-RD. Over half of the domain items are scored according to the presence of characteristic imaging manifestations, indicating the value of radiology in disease classification. In contrast to other published series [11–14, 17], this study evaluates 2019 ACR-EULAR classification criteria from a radiological perspective, offering subspecialty insight into the real-world applicability of these criteria.

In this study, 68.9% of patients were classified as IgG4-RD according to the 2019 ACR-EULAR classification criteria after scoring all domains. The sensitivity of the 2019 ACR-EULAR classification criteria varies across studies, ranging from 59.9 to 97.5% [11–14, 17], with higher sensitivities observed when the initial diagnosis was confirmed through biopsy [11, 17]. In our study, however, biopsy results were available only in 56.7% of patients. Of these, approximately half of the patients exhibited typical histological findings, and two-thirds met the IgG4 + immunostaining thresholds defined by the ACR-EULAR. Furthermore, nondiagnostic biopsy samples are commonly encountered in routine practice [1]. In the cohort of 51 patients classified as IgG4-RD, the radiology domain was high enough to establish the diagnosis in 39.2% of the patients, independent from other domains. Noteworthy, combining the radiology and serology domains led to the diagnosis of an additional 39.2% of patients. Consequently, approximately 80% of the patients fulfilled the diagnostic criteria without requiring biopsy results. Our results confirm the validity of the ACR-EULAR classification system, showing that histopathologic criteria may not be required for diagnosis if other domains' scores are met [9, 10].

IgG4-RD radiologic manifestations involving two or more organs were more frequently seen in patients who met the 2019 ACR-EULAR classification criteria than those who did not (84.3% vs 21.7%, *p* < 0.001). Our results corroborate previous observations by the ACR-EULAR investigators and Della-Torre et al. [9, 11, 14], demonstrating a significant correlation between the number of organs involved at presentation and the classification score. This also aligns with other studies that showed extrapancreatic involvement (Figs. 1 and 2) helps differentiate autoimmune pancreatitis from pancreatic cancer, which is a potential mimicker of IgG4-RD [18, 19].

Patients in our cohort were more likely to have abdominal imaging at baseline compared to head, neck and chest studies. The distribution of imaging studies is not unexpected since abdominal IgG4-RD manifestations are well-described [20, 21], and pancreatic manifestations are highly specific for diagnosis. However, this bias potentially underrepresents the number of patients with predominantly extra-abdominal IgG4-RD.

The high level of interobserver agreement in evaluating the salivary and lacrimal glands and abdominal organs is likely due to the highly specific imaging features of IgG4-RD in these organs, as seen in ultrasound for salivary/lacrimal glands [22] and CT/MRI for the pancreas [23]. For instance, to achieve the highest score of 11 points for the pancreatic IgG4-RD, diffuse pancreatic enlargement must be accompanied by the highly specific sign of a “capsule-like rim” (Fig. 2). However, it should be emphasised that this sign has low sensitivity, especially in focal form autoimmune pancreatitis [24]. In contrast, the lowest level of agreement was observed in the assessment of the chest, which may be attributed to reduced reader familiarity with the manifestations of IgG4-related lung disease, as well as the nonspecific imaging features of this condition in the lungs [25].

Although our dataset corroborates the feasibility of the 2019 ACR-EULAR classification criteria, it is worth considering a comprehensive elucidation of the imaging characteristics and formulating specific recommendations regarding the choice of imaging modalities for individual anatomical regions. For instance, CT and MRI have a high diagnostic accuracy in abdominal manifestations of IgG4-RD [23], while US can serve as a simple screening tool for the disease in salivary and lacrimal glands [26] (Fig. 3), especially due to its lack of radiation and high sensitivity and specificity [22]. Furthermore, advanced imaging techniques such as diffusion-weighted imaging (DWI), in conjunction with conventional MRI sequences, can be surrogate for diagnostic criteria due to its improved performance in detecting and characterizing lesions, specifically in IgG4-RD involving the pancreas [27–29] and kidney [30, 31]. DWI may be particularly useful to identify multi-organ involvement in IgG4-RD in the abdomen, where the confluence of pancreatic and renal manifestations could amplify diagnostic confidence [19, 32]. Noteworthy, the 2019 ACR/EULAR classification system prioritises highly specific radiologic criteria and does not assign scoring to several features acknowledged in clinical practice as indicative of IgG4-RD [9, 33]. Examples include the response of pancreatic and extrapancreatic disease to steroid therapy, as well as the presence of IgG4-related cholangiopathy accompanied by pancreatic atrophy, which may develop  as a consequence of preceding pancreatic disease [5, 8, 12, 20]. Furthermore, IgG4-related cholangiopathy is only scored if associated with typical pancreatic findings [9, 33].

## Limitations

The classification criteria were retrospectively applied to patients with existing IgG4-RD diagnoses and without matched controls or disease mimickers, we could not ascertain the numbers of true-negatives/false-positives to validate the specificity of criteria items. Our centre has extensive experience in diagnosing and treating IgG4-RD and is the hub institution for regional referrals. Correspondingly, test performance in our cohort may be less reproducible at smaller centers with low disease prevalence and low pre-test probability. Similarly, biopsies are less frequently performed at our centre; local clinician preference is to obtain diagnostic imaging to facilitate early diagnosis. The tendency towards non-invasive diagnosis is likely to impact subsequent univariate analysis. Other centres with greater reliance on or preference for histopathological confirmation of IgG4-RD may encounter different results regarding univariate analysis and classification criteria performance.

## Conclusion

Our study underscores the pivotal role of the radiology-related inclusion criteria of the 2019 ACR-EULAR classification system in diagnosing IgG4-RD. Nearly 80% of the patients within our cohort met the diagnostic criteria primarily based on radiology and serology domains, enabling potential non-invasive diagnosis of IgG4-RD. Multiple organ involvement at presentation, notably the pancreas and biliary system, was strongly associated with the classification of IgG4-RD. Our study demonstrated high inter-reader agreement in scoring the radiology domain, indicating a consistent interpretation of imaging findings.

## Data Availability

The datasets analysed during the current study are not publicly available due to local restrictions of data protection. In order to discuss the availability of the minimal dataset that would be necessary to interpret, replicate and build upon the findings reported in the article, the authors can be contacted.

## Abbreviations

ACR-EULAR: American College of Rheumatology and European League Against Rheumatism
CI: Confidence interval
CT: Computed tomography
HIPAA: Health Insurance Portability and Accountability Act
HPF: High-power field
IgG4: Immunoglobulin G4
IgG4-RD: Immunoglobulin G4-Related Disease
IQR: Interquartile range
MRI: Magnetic resonance imaging
REB: Research Ethics Board
RIS: Radiology Information System
ULN: Upper limit of normal
US: Ultrasound
